# Drs. Morgan and Clarke

**Published:** 1869-09

**Authors:** 


*Drs. Morgan and Clarke.*--In the editorial matter of the May No. of the *Journal* was published a selected article, from the *Dental Register*, written by Dr. W. H. Morgan, which contained a serious charge against Dr. F. Y. Clarke. This article referred to the action of Am. Dent. Association towards Southern Dentists, and accused Dr. Clarke of having in his possession at the Boston meeting, a secret service medal, obtained from Gen. Wilson, U. S. A., and exhibiting the same to certain members of the Association.

We are much gratified to be able to inform our readers, that at an interview which occurred between these gentlemen at the Atlanta meeting of the Southern Dental Association, the charge against Dr. Clarke was withdrawn by Dr. Morgan, the latter feeling that injustice had been unintentionally done the former.

From what occurred at the Boston meeting, Dr. Morgan felt that he was justified in making this charge, but as Dr. Clarke satisfied him that the medal in question was one of a totally different character, and that the remarks concerning it were uttered in jest, Dr. M. is now satisfied that injustice was unintentionally done Dr. Clarke.

In justice to both parties we make our readers acquainted with the true facts of the case by this explanation of the causes which led to the misunderstanding.

